# Medical students’ preferences towards learning resources and their study habits at King Abdulaziz University, Jeddah, Saudi Arabia

**DOI:** 10.1186/s13104-019-4052-3

**Published:** 2019-01-17

**Authors:** Tahir Jameel, Zohair Jamil Gazzaz, Mukhtiar Baig, Jawad Mansoor Tashkandi, Nasser Saleh Alharenth, Nadeem Shafique Butt, Ansar Shafique, Rahila Iftikhar

**Affiliations:** 1 Department of Medicine, Faculty of Medicine, Rabigh, King Abdulaziz University, Jeddah, Saudi Arabia; 2 Department of Clinical Biochemistry/Medical Educationist, Faculty of Medicine, Rabigh, KAU, Jeddah, Saudi Arabia; 3 Faculty of Medicine, Rabigh, KAU, Jeddah, Saudi Arabia; 4 Department of Community and Family Medicine, Faculty of Medicine, Rabigh, KAU, Jeddah, Saudi Arabia; 5 Department of Surgery, Faculty of Medicine, Rabigh, KAU, Jeddah, Saudi Arabia

**Keywords:** Textbooks, Study habits, Medical students

## Abstract

**Objective:**

The purpose of the present study was to explore why our medical students are avoiding the study of professional textbooks. We conducted this study from 10th March to 15th May 2017 at the King Abdulaziz University (KAU), Jeddah, to investigate their preferences towards learning resources, their study habits and correlation of academic achievements as a result of these trends. A questionnaire was provided as a web link. The participants of the study included medical students and data was analyzed on SPSS-Version 21.

**Results:**

A total of 347/500 medical students participated in the study. Among our participants, there were 123 (35.5%) males and 224 (64.6%) were females. Female students’ spent most of their time reading textbooks as compared to males (P-value = 0.001). Males mostly preferred the lecture handouts provided by their teachers. One-third of students admitted that, due to lack of a good grasp of English, they do not readily understand textbooks and consequently 67 (19.3%) students’ showed a lack of interest in textbooks. Majority of the males 103 (84%) spent twice a time (66 vs. 33%) watching television as compared to the females. WhatsApp and Facebook kept both the sexes busy in most of their spare time.

## Introduction

There is an explosion of information in the medical field due to extensive research in the medical sciences. Undergraduate medical students are expected to learn this vast amount of information in order to utilize it in their practice of medicine [1]. Many study resources, including textbooks, medical journals, concise booklets, lecture notes, online books, and Internet-related articles, are available to the medical students. All these accessible recourses help them to develop a good reading habit, which is an essential skill to thrive in the medical profession [2].

In medical institutions, the planning and execution of curriculum along with the related assessments are designed to promote learning habits in the students and to improve the ability of interpretation of available facts in clinical practice [3]. Attitude and approach to learning, which includes study habits and proper selection of study resources, have been known to be the key factors in predicting the student’s success in professional life [4].

In the prospects of Saudi Arabia, almost all the students get their early education in the Arabic language and when they enter in medical college, they face a different teaching and learning environment where all the books & lectures are to be comprehended in the English language. Literature search indicates that there is not enough research data available about the attitude of medical students towards their study resources, especially in this region. Internationally, several studies are available regarding this topic [1, 5, 6], and a few studies are also available from Saudi Arabia [7, 8] and in neighboring countries [8, 9].

The aim of the present study was to investigate medical students’ preferences towards learning resources and studying habits at KAU, Jeddah, Saudi Arabia.

## Main text

### Participants and measures

This cross-sectional study was conducted from 10th March to 15th May 2017 at the KAU, Jeddah. The study population included medical students of two campuses, i.e., the Faculty of Medicine Rabigh, and Jeddah girl campus of KAU, Jeddah. The participants of the study included students from second to final year, both male and female. The purpose of the study was explained to all the students and verbal consent was obtained from all the participants. Participation was voluntary. The ethical review committee of KAU reviewed and approved the project.

A self-administered questionnaire was provided online to all the students’ as a web link. The questionnaire included queries regarding the knowledge, attitude, evaluation of study habits, and how the students use available time in different recreational and time flies’ activities. The questions were formulated using the Likert scale in multiple questions. The facility of answering multiple options was available in most of the questions to increase the response rate. The questionnaire was distributed to 500 medical students of both genders and from the second year to the sixth year. For validity and reliability, the questionnaire was pretested on 30 students, Cronbach’s alpha was calculated, and a medical educationist and a senior professor did the content validity. The questionnaire was modified according to their suggestions.

### Data analysis

The sample size was computed by using RAOSOFT sample size calculator, taking 5% margin of error, 95% confidence level, 2200 population size and by assuming 50% of the student’s study textbooks. The calculated sample size was 328, but by considering students’ non-response rates, we collected more samples. The data was analyzed on SPSS-Version 21. The qualitative data was expressed in frequencies and percentages.

## Results

A total of 347/500 medical students responded. The response rate was 72%. Among our participants, there were 123 (35.5%) males and 224 (64.6%) females. Only 17 (4.9%) students were married.

Female medical students spent most of their time reading textbooks as compared to males (*P*-*value *= .001). Similarly, opinion about the essential version of basic textbooks was entirely different in both groups (*P*-*value* = .000) (Table 1). Online resources, such as general websites, online versions of textbooks, online journals, and medical websites were favored more by the female medical students, but no significant correlation was observed among them (*P*-*value *= .826, .101, .625, respectively).

**Table 1:** Comparison of gender preferences about study resources (students had the choice to select more than one item)

Statements	Gender		P-value
	Male	Female	
	N (%)	N (%)	
Medical textbook			
Very rarely	31 (52.5)	28 (47.5)	.001
Occasionally	45 (34.1)	87 (65.9)	
Never	11 (57.9)	8 (42.1)	
Most of time	33 (29.2)	80 (79.8)	
Always	3 (12.5)	21 (87.5)	
Essential version of a basic medical text book			
Very rarely	33 (39.3)	51 (60.7)	.000^*^
Occasionally	46 (34.6)	87 (65.4)	
Never	16 (72.7)	6 (27.3)	
Most of time	19 (22.1)	67 (77.9)	
Always	9 (40.9)	13 (59.1)	
Online sources			
Very rarely	19 (52.8)	17 (47.2)	.139
Occasionally	27 (32.5)	56 (67.5)	
Never	8 (47.1)	9 (52.9)	
Most of time	39 (31.5)	85 (68.5)	
Always	30 (34.5)	57 (65.5)	
Online version of textbook			
Very rarely	23 (34.3)	44 (65.7)	.826
Occasionally	36 (31.9)	77 (68.1)	
Never	16 (41)	23 (59)	
Most of time	34 (38.2)	55 (61.8)	
Always	14 (35.9)	25 (64.1)	
Online journal article			
Very rarely	29 (27.4)	77 (72.6)	.101
Occasionally	34 (34.3)	65 (65.7)	
Never	25 (37.3)	42 (62.7)	
Most of time	26 (49.1)	27 (50.9)	
Always	9 (40.9)	13 (59.1)	
Medical websites			
Very rarely	17 (43.6)	22 (56.4)	.652
Occasionally	24 (30)	56 (70)	
Never	9 (39.1)	14 (60.9)	
Most of time	45 (34.6)	85 (65.4)	
Always	28 (37.3)	47 (62.7)	
Pocketbooks			
Very rarely	35 (44.3)	44 (55.7)	.001
Occasionally	25 (23.8)	80 (76.2)	
Never	37 (51.4)	35 (48.6)	
Most of time	19 (27.5)	50 (72.5)	
Always	7 (31.8)	15 (68.2)	
Journal articles (print version)			
Very rarely	24 (23.3)	79 (76.7)	.008
Occasionally	26 (39.4)	40 (60.6)	
Never	39 (35.8)	70 (64.2)	
Most of time	22 (45.8)	26 (54.2)	
Always	12 (57.1)	9 (42.9)	
Lecture handouts			
Very rarely	8 (33.3)	16 (66.7)	.001
Occasionally	16 (26.2)	45 (73.8)	
Never	12 (70.6)	5 (29.4)	
Most of time	35 (27.1)	94 (72.9)	
Always	52 (44.8)	64 (55.2)	
Test preparation textbooks			
Very rarely	23 (35.9)	41 (64.1)	.687
Occasionally	40 (36.4)	70 (63.6)	
Never	23 (36.5)	40 (63.5)	
Most of time	18 (28.1)	46 (71.9)	
Always	19 (41.3)	27 (58.7)	
Lecture notes taken in the class			
Very rarely	15 (34.1)	29 (65.9)	.230
Occasionally	18 (28.6)	45 (71.4)	
Never	8 (47.1)	9 (52.9)	
Most of time	36 (31.3)	79 (68.7)	
Always	46 (42.6)	62 (57.4)	

In response to a question about the usefulness of various types of reading materials, most of the medical students favored lecture notes or handouts, online sources, and medical websites. The medical textbook was mentioned by 160 (46.1%) as the most useful source for studies.

According to the students, they spent most of their time in studying either their lecture notes prepared during the class or their lecture handouts provided by the teachers (*P value* = .005 and .001, respectively). Only 22% of the students commented that they studied their textbook in most of their available time.

Around one-third (119, 34.3%) of students admitted that, due to their poor grasp of English, they do not easily understand teaching material in textbooks written in English and about 67 (19.3%) showed a lack of interest in textbooks written in English. About 244 (70%) of them had the excuse of a lack of time for reading the textbook because of other commitments.

**Fig. 1:**
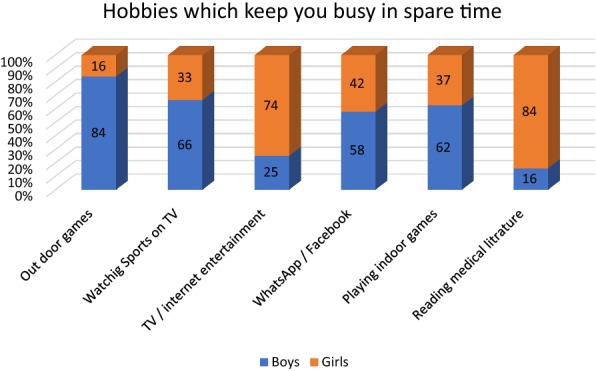
Participants’ hobbies, which keep them busy in spare time

In response to the question about the hobbies, 103 (84%) of male students and 35 (16%) of girls mentioned outdoor games as their hobby. Multimedia applications such as WhatsApp and Facebook kept both the sexes busy in most of their spare time, but the boys dominated by 55% vs. 42% (Fig. 1).

A significant finding was that most of the students (287, 82%) believed problem-based learning (PBL) and other related class activities, where the students must study to find out an answer, promote textbook reading in them to a great extent. Two hundred and seventy-five (80%) of the students admitted that, in almost all the study guides of different modules, textbooks were recommended for studies. A similar proportion of students believed that the availability of the latest textbooks was not a problem for them as they were available in the college library in sufficient numbers.

**Fig. 2:**
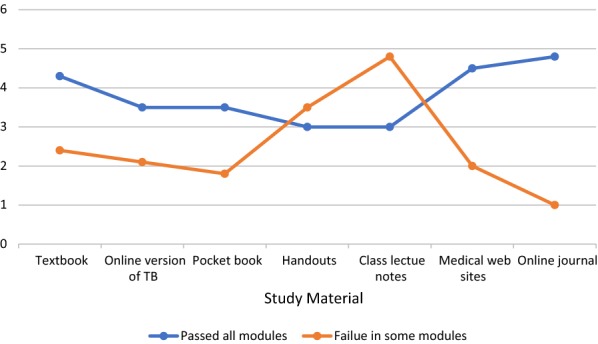
Correlation of success in previous module and preferences of study material

While responding to the questionnaire, only 33 (9.5%) participants admitted failure in previous years in any module. The remainder of 314 (90.5%) passed in all their previous examinations. We correlated the selection of study material with success in previous modules and, quite interestingly, the students that passed in all previous modules showed interest in reading medical textbooks in a much higher proportion compared to those who had to face failure in any of their previous modules (P-value = 0.005). The students passing in all their previous exams also revealed their liking for online versions of textbooks, pocketbooks, medical websites, and online journals whereas the other group had more liking for lecture handouts and lecture notes from their classes (*P*-*value * = .001) (Fig. 2).

## Discussion

Medical learning is a complex procedure and there is a stressing need of an individual commitment, developing reading habits and building up of communication skills. Reading habit is essential in clinical skills; it enhances knowledge and thinking process regarding day-to-day clinical challenges [10]. Medical teaching faculty has a key role in providing a supportive environment to the students in acquiring essential qualities for future responsibilities in their professional life [11].

In our research, we assessed our students in terms of their study habits, selection of material for professional studies, their likes and dislikes while selecting the available resources, average time of professional studies in a day, their hobbies in their spare time, various teaching activities which compel them to textbook consultation and the attitude of their teachers in promoting the habit of textbook reading in them.

Around 80% of girls mentioned their preference for textbook reading whereas only a small group of male students (29.2%) mentioned textbook as their preferred reading source. Similar results were observed in a study, where the girls preferred textbook much more than the boys [12]. Other sources of professional studies such as online textbooks, handbooks, medical websites, and other Internet sites, attracted the female students much more than the male students (72.5 vs. 27.5%). A recent study in Saudi Arabia proposed that boys have more outdoor attractions as compared to girls, as the girls are not allowed to enjoy outdoor activity freely so naturally, they have more time for professional studies than the boys [10]. A study from the KAU reported that most of the medical students have Medical Apps in their smart devices and most of the students used smartphones to search for medical information and get medical news [13].

In KSA, newly admitted medical students study English in the first year (foundation year) along with basic sciences like biology, chemistry, and physics. Medical training starts at the start of the second year. Most of the students are not very confident in English in their initial years [14, 15]. A noticeable number of our students admitted that they could not understand the language of textbooks and almost 20% rather disliked textbook reading because of the same problem. A similar observation has been mentioned in a couple of studies on medical students in Saudi Arabia, that proficiency in the English language is the main reason for avoidance of textbook reading in the early years of medical education [15, 16].

Availability of Internet facilities at the individual level has many advantages; many applications have been developed to help the medical student and younger physicians in their daily clinical challenges [13], Applications like WhatsApp, Twitter and Facebook are so absorbing and time-consuming that one can see students being busy with them even while active teaching is going on. Halboub et al. have mentioned the effect of these applications in drastically lowering the GPA of medical students [17]. According to a published report on the Arab world, on average a young individual spends 28% of his time daily on social media [18]. An elaborate study regarding social media addiction among health science students revealed that almost all the students in the study were extensively using YouTube, Facebook and WhatsApp [19].

Our students pointed out that certain teaching modalities like Problem-based learning (PBL) and case-based learning (CBL) compelled them to open the textbooks to find specific answers related to clinical situations. A recent Saudi study has also suggested the implementation of an outcome-based curriculum with integrated efforts to promote textbook consultation among students [20].

In a recent study, it was pointed out that student success is related to many factors including the habit of textbook reading, proper note taking in class and concentrating on deep concepts regarding the subject [21]. Our students, having developed the study habits of reading textbooks through hard copy or by an online version, pocketbooks and medical websites, were successful in the modular exams as compared to a minority of students (9.5%) who relied mainly on lecture notes and handouts. The educational environment plays a vital role in students’ learning. A study from our college evaluated the educational learning environment by DREEM Inventory and reported that, at the Rabigh and Jeddah campuses of KAU, the students perceived a positive educational environment [22].

We strongly suggest that there is a need to change the assessment tools because students always prefer the stuff that is compatible with their assessment. Several studies have pointed out that by avoiding the tools that assess higher cognitive levels may compel the students to adopt superficial learning approaches and just memorization [23, 24]. A study pointed out that there is a need to use such assessment tools that improve students’ deep learning, for example, a concept map, and they pointed out that for such tools deep understanding of the topic is mandatory and, that is not possible without deep study of the textbooks [25]. The student’s response to studying from the textbooks is not very encouraging. There is a need for changes in the curriculum at school and college level, and the science subjects should be taught in English. Moreover, the teaching strategies and assessment tools need modifications to promote textbook reading habit in our medical students. We recommend further studies in this regard also from other institutes to explore study habits in other regions of Saudi Arabia.

## Limitations

The present study has a few limitations. The sample size is not large enough. This is a questionnaire-based study so individual responses and personal feeling of the students cannot be acknowledged. Moreover, the learning styles of the participants were not determined. It needs to be covered in a separate project.

## Abbreviations

KAU: King Abdulaziz University
PBL: problem-based learning
CBL: case-based learning
